# The paradoxical extinction of the most charismatic animals

**DOI:** 10.1371/journal.pbio.2003997

**Published:** 2018-04-12

**Authors:** Franck Courchamp, Ivan Jaric, Céline Albert, Yves Meinard, William J. Ripple, Guillaume Chapron

**Affiliations:** 1 Ecologie, Systématique and Evolution, Univ. Paris-Sud, CNRS, AgroParisTech, Université Paris-Saclay, Orsay, France; 2 Department of Ecology and Evolutionary Biology, University of California, Los Angeles, Los Angeles, California, United States of America; 3 Center for Tropical Research, Institute of the Environment and Sustainability, University of California, Los Angeles, Los Angeles, California, United States of America; 4 Biology Centre of the Czech Academy of Sciences, Institute of Hydrobiology, Na Sádkách, České Budějovice, Czech Republic; 5 Leibniz-Institute of Freshwater Ecology and Inland Fisheries, Müggelseedamm, Berlin, Germany; 6 Institute for Multidisciplinary Research, University of Belgrade, Kneza Viseslava, Belgrade, Serbia; 7 Université Paris Dauphine, Paris Sciences & Lettres Research University, CNRS, LAMSADE, Paris, France; 8 Global Trophic Cascades Program, Department of Forest Ecosystems and Society, Oregon State University, Corvallis, Oregon, United States of America; 9 Department of Ecology, Grimsö Wildlife Research Station, Swedish University of Agricultural Sciences, Riddarhyttan, Sweden

## Abstract

A widespread opinion is that conservation efforts disproportionately benefit charismatic species. However, this doesn’t mean that they are not threatened, and which species are “charismatic” remains unclear. Here, we identify the 10 most charismatic animals and show that they are at high risk of imminent extinction in the wild. We also find that the public ignores these animals’ predicament and we suggest it could be due to the observed biased perception of their abundance, based more on their profusion in our culture than on their natural populations. We hypothesize that this biased perception impairs conservation efforts because people are unaware that the animals they cherish face imminent extinction and do not perceive their urgent need for conservation. By freely using the image of rare and threatened species in their product marketing, many companies may participate in creating this biased perception, with unintended detrimental effects on conservation efforts, which should be compensated by channeling part of the associated profits to conservation. According to our hypothesis, this biased perception would be likely to last as long as the massive cultural and commercial presence of charismatic species is not accompanied by adequate information campaigns about the imminent threats they face.

One of the difficulties faced by endangered species conservation efforts is the lack of a strong public support and mobilization. Whereas the biodiversity decline shows no sign of abatement, public mobilization has not scaled up with the severity of this crisis. For example, 20 million Americans took to the streets to demonstrate on the first Earth Day in 1970, but similar levels of mobilization have not been seen in the 21st century. This surprising discrepancy between the need for global mobilization to avert species extinction and the lack thereof might be due in part to the disconnection of the general public from nature [1], because many endangered species and ecosystems are of limited appeal for the broad public. Here, we argue that the problem stems from deeper roots, because even the most charismatic wild animals suffer from the same predicament. We show that, paradoxically, the most charismatic species remain severely endangered, and rather unknowingly so by the general public, a situation that has dramatically worsened over the last decades despite massive cultural and commercial presence.

The concept of charismatic species is pervasive in the conservation literature and refers to species attracting the largest interest and empathy from the public [2]. As a consequence, charismatic species are often considered as privileged by having enjoyed the bulk of conservation efforts [3]. Therefore, the conservation of charismatic species is often taken for granted, and accordingly the literature emphasizes the need to go beyond charismatic species to conserve more discrete ones [4] and even to shift the conservation focus towards units that are more integrative and less visible to the broad public, such as ecosystems or ecosystem functions [5]. Using four different methods, we established the ranking of the 10 most charismatic species for the public and reviewed their conservation status and the public knowledge of it. We unveil that the conservation status of the ten most charismatic species is grave, while the public ignores it. We surmise that this “beloved but ignored” paradox may stem from a mismatch between the virtual presence and natural presence of these particular species. We argue that the representations of charismatic species in commercial, artistic, and cultural outlets act as virtual, abundant populations competing for public attention against real threatened populations. The competitive advantage of virtual populations reinforces the perception that natural populations are not threatened and may paradoxically lessen the necessary conservation efforts and consequently accentuate the risk of extinction of these species most cherished by the general public. We propose a mechanism whereby these virtual populations would not compete against threatened species but instead benefit them through a payment mechanism represented by fees for rights of use for commercial purposes.

## Identifying the 10 most charismatic animals

Although species charisma is increasingly used in conservation biology [2], this concept has never been operationalized, and which species the public considers the most charismatic has not been established. We collected data from four complementary sources to quantify the charisma of species for the Western public (see S1 Text for details): (i) an online large-scale survey (*n* = 4,522); (ii) a questionnaire given to primary schoolchildren of France, Spain, and England (*n* = 224); (iii) a survey of the animals displayed on the websites from zoos in the 100 largest cities in the world; and (iv) a survey of the animals featured on the covers of animated movies produced by Disney and Pixar (see S1 Text). The first two sources represented direct questions to the public about which species they perceived as charismatic, while for the other two, we worked under the assumption that the species displayed on zoo websites and movies would be selected by communication experts based on their appeal to the public. The survey on pupils was intended to complement the internet survey for which children below 15 years old represented only 0.9% of the 4,522 respondents. Collectively, these data provided a coherent list that can be considered representative of animals regarded by the Western public as being the most charismatic. We call them animals instead of species, because taxonomic precision to the species level for public knowledge was possible for none of the four sources and, among the top 10 animals cited, 2 represent more than one species. Indeed, elephants represent three species, while gorillas represent two species; we will thus hereafter mention 10 animals or 13 species. The compiled list of the 10 animals considered the most charismatic by the public was in this order (S1 Fig): the tiger (*Panthera tigris*), the lion (*P*. *leo*), the elephant (*Loxodonta africana*, *L*. *cyclotis*, and *Elephas maximus*), the giraffe (*Giraffa camelopardalis*), the leopard (*P*. *pardus*), the panda (*Ailuropoda melanoleuca*), the cheetah (*Acinonyx jubatus*), the polar bear (*Ursus maritimus*), the gray wolf (*Canis lupus*), and the gorilla (*Gorilla beringei* and *G*. *gorilla*).

## Severe endangerment of the most charismatic species

**Fig 1:**
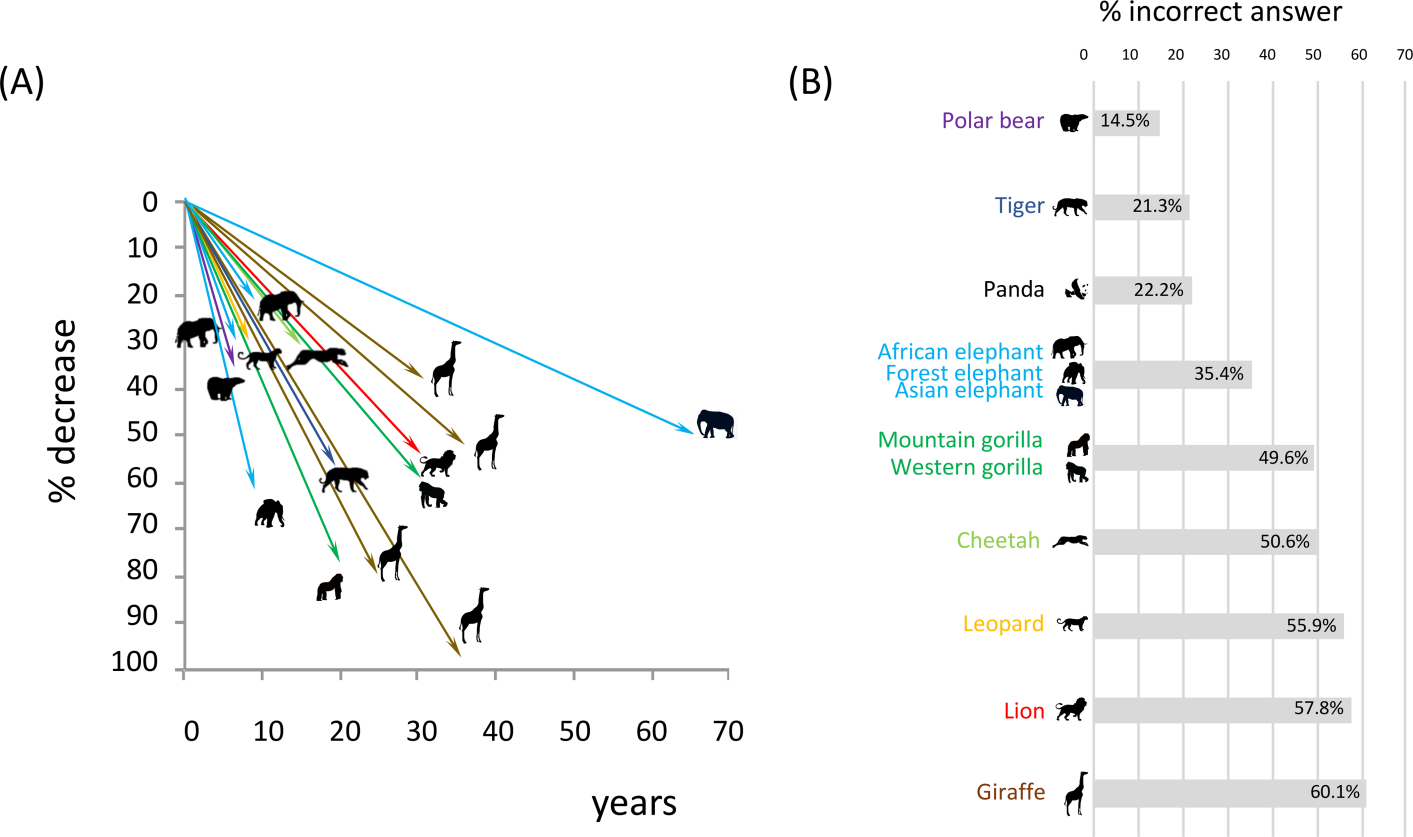
(A) Recent, dramatic declines of the most charismatic animals. Time, but not date, is taken into account, explaining why all trajectories have the same origin. Long, steep lines indicate a large decline at a high rate. Icons represent populations. Wolf is not represented and 4 subspecies of giraffes are represented. The declines are tigers: over 55% in the last 20 years [7]; African lions: 54% over the last three decades [40]; African elephants: over 20% over less than 10 years [41]; savannah elephants: over 30% between 2007 and 2014 [42]; Central African forest elephants: 62% between 2002–2011 [14]; Asian elephant: over 50% in 65 years [15,16]; giraffes: 38% in the last 30 years [17]; Masai giraffes (*Giraffa camelopardalis tippelskirchi*): 52% in 35 years [17]; reticulated giraffes (*G*. *c*. *reticulata*): 80% in 25 years [17]; Nubian giraffes (*G*. *c*. *camelopardalis*): 97% in 35 years [17]; leopards: over 30% in 8 years [19]; cheetahs: over 30% in the last 15 years [24,25]; southern Beaufort Sea polar bears: 63% between 2004 and 2010 [43,44]; Grauer’s gorillas: 77% in less than 20 years [45]; Western lowland gorillas: nearly 60% in 30 years [30]. (B) Percentage of incorrect answers to the question, “Is this species endangered,” reflecting biased knowledge of conservation status of the most charismatic species. See text for details.

Although conservation efforts are indeed probably disproportionately focused on them, these 13 species are nevertheless in a dire conservation status (Box 1, Tables 1 and S1). Except for the gray wolf, all the species are either Vulnerable, Endangered, or Critically Endangered [6]. Furthermore, most of the species that are classified within lower threat categories, such as Vulnerable, are considered as such based on global and outdated assessments, masking important discrepancies between more threatened populations or subspecies. Although conservation biology has been particularly active the last three decades, dramatic declines have taken place over the same period, with losses often exceeding half of the entire species’ population in an extremely short time (Fig 1A). One interesting observation is that direct killing constitutes one of the principal causes of endangerment, a surprising finding for the 10 most charismatic animals (see S2 Text). Moreover, population estimates are generally provided as global numbers, masking the fact that the number of breeding animals is often much lower and that the global population corresponds to many disconnected populations, many of which are too small to be viable (see S2 Text). Demographic studies of minimum viable population (MVP) show insufficient population sizes to expect high survival probabilities in the short term if strong conservation measures are not taken rapidly (Table 1).

**Box 1. The jeopardized future of the 10 most charismatic species.**

- Tiger—total abundance estimated at less than 7% of their historic numbers [7]. Three subspecies (Bali tiger, *P*. *tigris balica*; Javan tiger, *P*. *t*. *sondaica*; and Caspian tiger, *P*. *t*. *virgata*) are now extinct, and another one (the South China tiger, *P*. *t*. *amoyensis*) is considered as possibly extinct [8,9].
- Lion—declining almost everywhere in Africa, with populations estimated to be at less than 8% of historic levels [10,11]. In Eurasia, with the exception of the only remaining population of about 175 individuals of *P*. *leo persica* in India, all lions are now extinct [12].
- Elephant—the African savannah elephant never recovered from the 20th century massive poaching levels and are believed to remain at less than 10% of their historic numbers [13]; the African forest elephant declined in a mere 9 years (2002–2011) by 62%, with about 30% corresponding range contraction [14]; the Asian elephant lost 85% of historic range, and almost half of the remaining 15% is both fragmented and heavily impacted by an ever increasing human population [15,16].
- Giraffe—previously classified as Vulnerable because it was assessed as a single species [17]. Three of the four newly identified species [18] have declined by 52%–97% in the last 35 years and are likely to be uplisted by IUCN [17].
- Leopard—classified as a Vulnerable species because the 9 subspecies are altogether abundant and widely distributed [19]; however, 3 are Critically Endangered, 2 are Endangered, 2 are recommended for uplisting to Critically Endangered and Endangered, and 2 are Near Threatened [20]. Leopards have already lost as much as 75% of their habitat range, and 6 out of the 9 subspecies occupy a mere 3% of the remaining range [20].
- Panda—with fewer than 2,000 remaining individuals, distributed within 33 subpopulations and scattered on less than 1% of its historic range, its future remains particularly uncertain [21], especially in light of climate change, predicted to reduce most of its bamboo habitat [22,23].
- Cheetah—occupies only 9% of its historic range in Africa, being extirpated from 29 countries on the continent [24], while the Asiatic subspecies *Acinonyx jubatus venaticus*, numbering fewer than 100 individuals, is listed as Critically Endangered [25].
- Polar bear—lack of population abundance and trend estimates; 9 out of the 19 known populations are of unknown status. It is considered severely impacted by climate change and related sea ice decline [6]. The few populations with available data show drastic population declines (see Fig 1).
- Wolf—once the world’s most widely distributed large predator, it has now lost about one third of its original range, becoming extinct in much of Western Europe and the United States and being endangered in several other regions [26].
- Gorilla—of the 4 gorilla subspecies, 2 are limited to a few hundred individuals in small and highly fragmented populations [27,28], while the 2 others have lost most of their numbers in about 20 years [29,30].

**Table 1:** Status and trends of the 10 most charismatic animals. See S1 Text for the calculus of variables. African forest elephants have been distinguished from savannah elephants when information was available.

Species			IUCN	Demography			Habitat			
Species		Taxonomic fractionation	Status	Estimated population size	Current trend	Percent MVP (pop/#patch)/MVP	Percent historical range	Percent range protected	Percent “viable” habitat	Fragmentation (#patch)
Tiger	*Panthera tigris*	9 subspecies	EN	3,159	ꜜ Decr.	30	<6^h^	36	76	>54^p^
Lion	*P*. *leo*	2 subspecies	VU	20,000^a^	ꜜ Decr.	155	17^i^	82	84	67^q^
Elephant	*Loxodonta Africana*	3 species	VU	500,000^b^	ꜛ Incr.*	1,431	19.9^b^	57	83	70^r^
	*L*. *cyclotis*		/	<100,000^c^	ꜜ Decr.	231	<25^c^	57	83	70^r^
	*Elephas maximus*		EN	47,000^b^	ꜜ Decr.	93	15^j^^,^^k^	30	67	>138^s^
Giraffe	*Giraffa camelopardalis*	4 species, 9 subspecies	VU	80,000^b^	ꜜ Decr.	714	11.3^b^	57	77	>66^t^
Leopard	*P*. *pardus*	9 subspecies	VU	Unkn.	ꜜ Decr.	/	25^l^	34	7	289^l^
Panda	*Ailuropoda melanoleuca*	/	VU	1,864^d^	ꜛ Incr.	23	<1^m^	62	94	33^m^
Cheetah	*Acinonyx jubatus*	9 subspecies	VU	7,000^e^	ꜜ Decr.	45	9^n^	40	51	29^e^
Polar bear	*Ursus maritimus*	/	VU	Unkn.	Unkn.	/	Dyn	12	24	19^u^
Wolf	*Canis lupus*	12 subspecies	LC	Unkn.	→ Stable	/	About 66^o^	14	58	/
Gorilla	*Gorilla beringei*	2 species, 4 subspecies	CR	3,800+880^f^	ꜜ Decr.	388	/	51	93	2^f^; 4^f^
	*G*. *gorilla*		CR	300+150,000^g^	ꜜ Decr.	5,330	/	24	89	13^v^^,^^w^; /

References:

^a^[31]

^b^[32]

^c^[14]

^d^[33]

^e^[25]

^f^[29]

^g^[30]

^h^[34]

^i^[35]

^j^[32]

^k^[16]

^l^[20]

^m^[21]

^n^[24]

^o^[26]

^p^[9]

^q^[12]

^r^[36]

^s^[37]

^t^[17]

^u^[38]

^v^[27]

^w^[39]

“/” means “no data available.”

* IUCN assessment of 2008; shown to be decreasing since

Abbreviations: #patch, number of patches; CR, Critically Endangered; Decr., decreasing; Dyn, dynamical; EN, Endangered; Incr., increasing; IUCN, International Union for the Conservation of Nature; LC, Least Concern; MVP, minimum viable population; Unkn., unknown; VU, Vulnerable.

**Fig 2:**
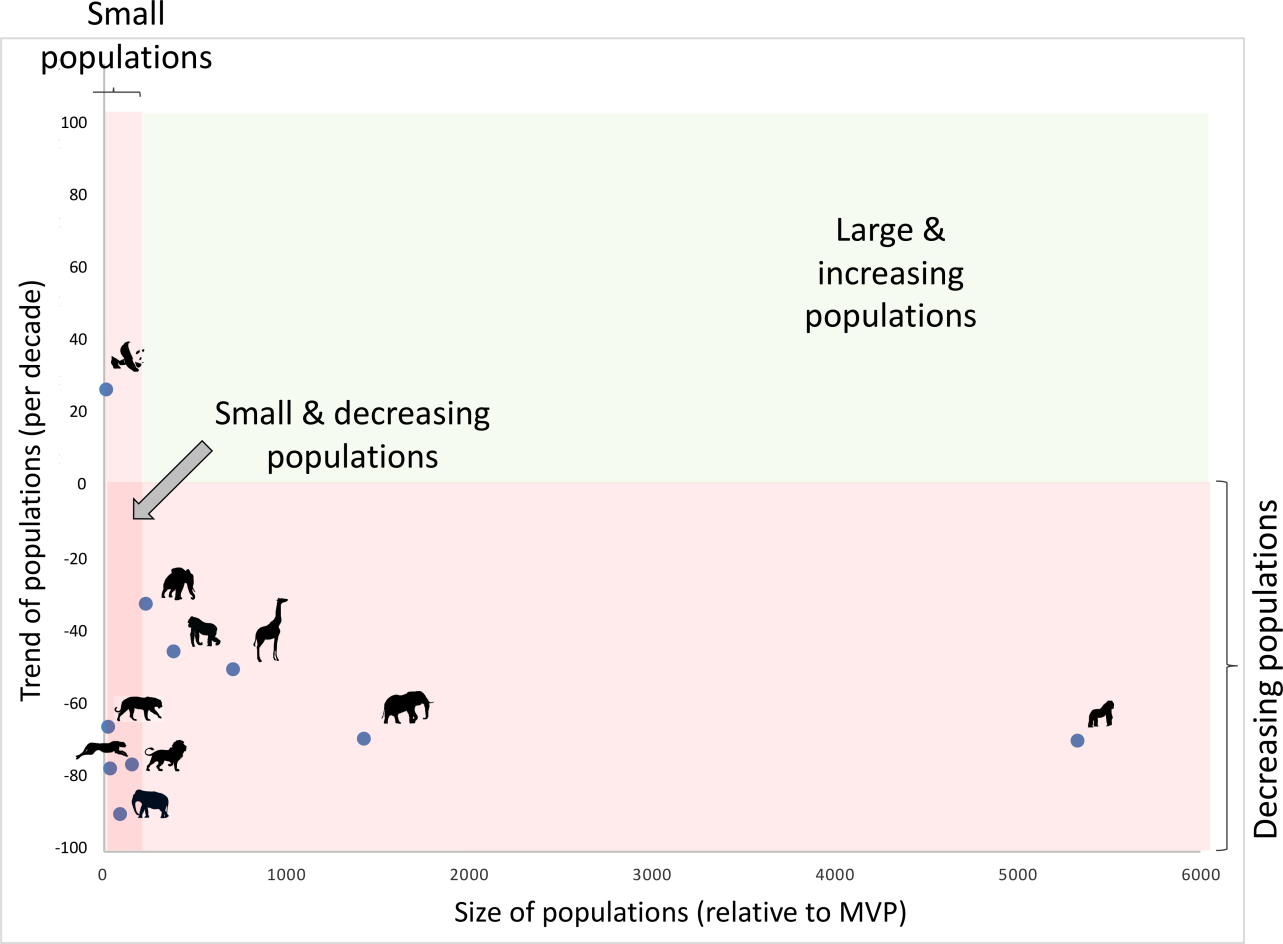
Population sizes and trends of the 10 animals. Trends are calculated per decade, based on the data presented in Fig 1 (and on the latest IUCN assessment for the panda [33]). Average population size is calculated conservatively as the overall population size divided by the number of isolated patches (see Table 1 for data and references). Resulting size is shown relative to the MVP size, as calculated for these species in recent syntheses [46]. Note that previous syntheses provide more pessimistic data, with MVPs one order of magnitude higher [47,48]. Icons correspond to names in Fig 1. Note that no overall population sizes are available for polar bears, wolves, and leopards, and no trends are available for wolves, while data were available for forest elephants, which we have here distinguished from savannah elephants. IUCN, International Union for the Conservation of Nature; MVP, minimum viable population.

The severe decline of species ranges is even more of a problem because all these species are large mammals requiring extended individual home ranges and correspondingly wide population distributions (S1 Table). As a result, most species suffer simultaneously from the declining and the small population paradigms (Fig 2). In the few remaining habitats of those 10 charismatic animals, the percentage of range in protected area is insufficient (41 ± 20, mean ± SD, Table 1) and the percentage of land under human pressure remains substantial (33 ± 28, Table 1). An aggravating factor for all populations is the severe fragmentation, by both the number of patches (71.3 ± 81.1, Table 1) and the surface/edge ratio (0.069 ± 0.12, S1 Table).

## Public ignorance of threatened status

Perhaps even more noteworthy than the poor conservation status of the animals that people cherish the most is our finding of the lack of awareness of the public on this matter. First, with two of our approaches to identify the most charismatic species (internet survey and pupil poll), we asked respondents whether they would associate each species with being “Endangered.” This attribute was selected less often than randomly, and less often than expected if the conservation status was known, suggesting that the public is often unaware that the animals they deem charismatic are threatened with extinction. Second, we conducted a targeted survey among students of the University of California, Los Angeles campus, in 2015. Ninety-six students were asked during individual interviews whether or not the 10 animals listed by the 4 sources were threatened; results (Fig 1B) show similarly that the public, even when represented by scientifically educated respondents, is often unaware of the dire conservation status of most of these species. On average, one in two persons was incorrectly assessing the endangerment of these animals, be it the general public or the supposedly more educated students of a world-class university. Exceptions are pandas, tigers, and polar bears, for which communication efforts may have borne their fruits in this regard—the first one being widely recognized as a global conservation icon and the two others as flagship species for traditional medicine and climate change impacts. Overall, these two lines of evidence suggest that these ten animals are not perceived as charismatic because of their conservation status, which is often not known. Conversely, these animals may be assumed to be abundant because of their omnipresence in our culture, as they are seen everywhere—in zoos and toys, on small and large screens, on advertisements and books alike. We emphasize that the gap between conservation status awareness and actual status should be especially unlikely in the most charismatic species, due to the high level of public attention they receive.

## Competition between real and virtual populations

Despite their poor conservation status, these species are omnipresent in our modern societies. A good illustration is in the advertising realm. Charismatic animals are often prime candidates for product marketing purposes or general cultural consumption. For example, 48.6% of all non-teddy bear plush animal toys sold on Amazon (US) were one of the ten animals, suggesting high likelihood that a majority of children has/had at least one of them as a stuffed companion during their childhood. Similarly, the number of “Sophie la girafe” baby toys sold in France (800,000 in 2010) exceeds the number of babies born [49] and is over 8 times more than the number of actual, living giraffes in Africa [17].To further support our idea, we asked 42 volunteers to document every encounter with one of the 10 species in “virtual” populations (commercial, artistic, cultural, in zoos, books, magazines, on objects, on logos, on television, etc.) during 7 consecutive days. All volunteers lived in France, with a combination of rural and urban environments, living with or without television, with or without interest in animals, etc. On average, they encountered up to 31 individuals of each of the 10 species, which corresponds for each person to several hundred total encounters per month (S2 Fig). For example, the volunteers saw an average of 4.4 lions a day, meaning that people see on average two to three times as many “virtual” lions in a single year than the total population of wild lions currently living in the whole of West Africa. This reinforces our idea that the ubiquity of “virtual” species may be hindering the perception of rarity of these animals.

These species are therefore ubiquitous in our culture through what could be called virtual populations. The public perception of the conservation status of these species appears to reflect virtual populations rather than real ones. This is not surprising, because most people will only see wild animals in virtual populations. We suggest that the abovementioned mismatch between perceived and real conservation status may be due to the fact that people base their perception of these species on their virtual rather than real populations. It is unfortunately difficult to unambiguously demonstrate a causal relationship between the overabundance of virtual species and the biased perception of their actual endangerment, mostly because causes of ignorance are always multiple and difficult to isolate and unconditionally tie in.

**Fig 3:**
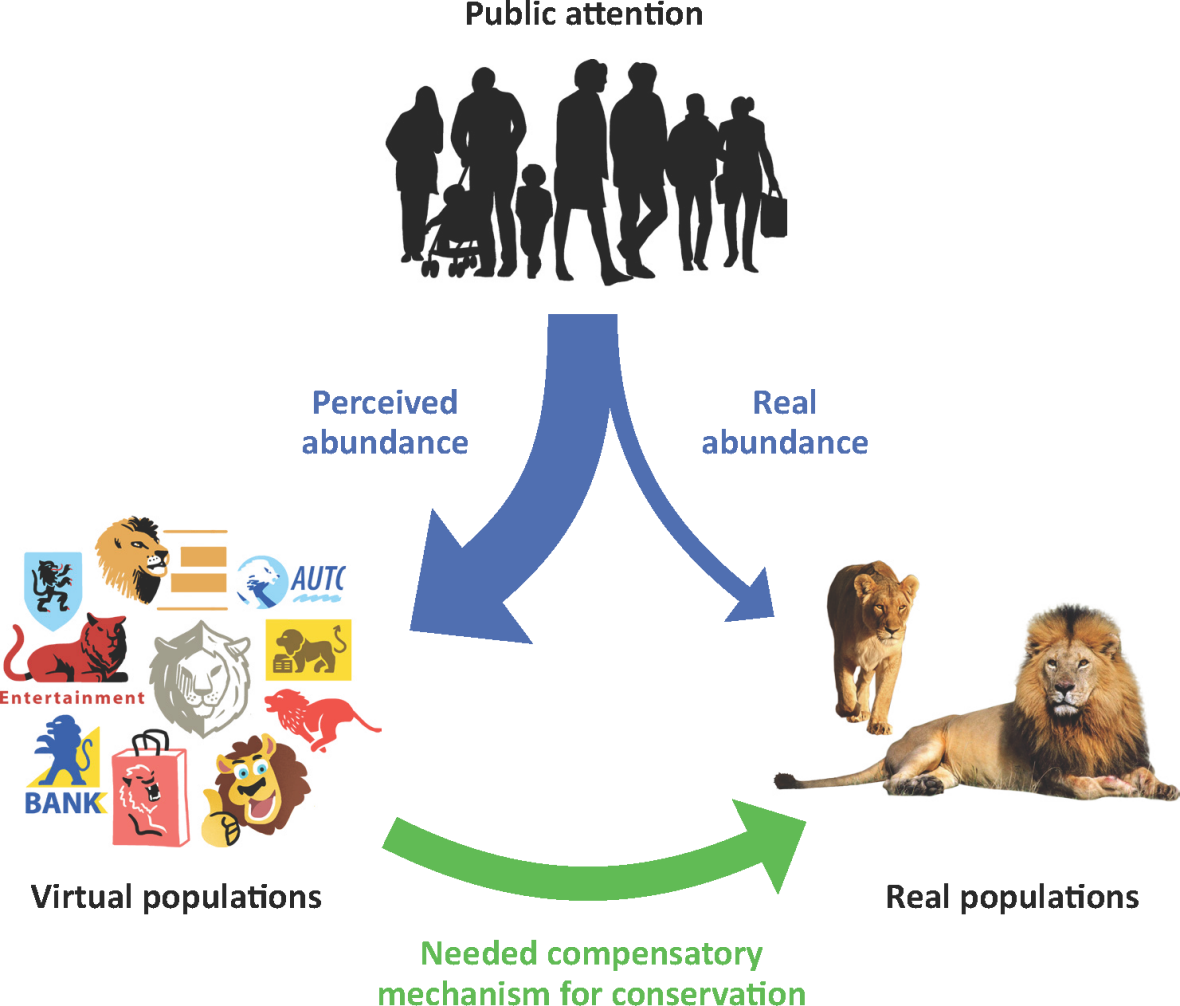
Illustration of the competition hypothesis between virtual populations (here represented by iconic logos of commercial companies) and natural populations (here represented by clip art of real animals), whereby abundance of virtual animals in culture alters the perception of actual rarity in real animals and, therefore, the perceived need for their conservation. A compensatory mechanism is needed to restore adequate conservation funding. *Credit for logo illustration and human silhouettes*: *Mathieu Ughetti*.

This mechanism amounts to an intraspecific competition mediated by human perception, in which abundant but virtual populations outcompete for human attention the real but threatened populations (Fig 3). The perceived extinction risk, which is low, as it is influenced by highly abundant, virtual populations, masks the real, high extinction risk. As humans would expectedly strive to prevent extinction of, at the very least, the 10 animals they cherish the most, the fact that they make decisions (or refrain from making any) based on perceived risk rather than the real one [50] would likely prevent conservation efforts from getting the necessary support.

## Turning competition into cooperation for conservation

One challenge for the success of conservation biology may therefore be how to transform these omnipresent but virtual populations from a liability to an asset for real populations. By using animals in their marketing, some products and brands gain a competitive market advantage, but the induced damages of contributing to the creation of competing, virtual populations are never taken into account and never compensated for. According to economic theory, such damages are “externalities,” and they must be “internalized” through institutional arrangements and/or payment mechanisms compelling companies to take responsibility for the damages they cause—albeit inadvertently [51]. Currently, companies do not pay a fee to use lions for their branding but, as we hypothesize, may unknowingly and indirectly weaken conservation support by contributing to a mistaken perception that lions are abundant, akin to a competition for attention from the public. Linking the use of threatened animal representations for commercial use to payment to conservation efforts could contribute to turning competition into cooperation between virtual and real populations. This logic of payment for rights to use is not different to, e.g., merchandising of derivative products: a clothes company needs to pay a fee to display the photo of a celebrity, of the illuminated Eiffel tower, or of the English Premier League logo on its products. That fee is paid to the brand copyright holder, in that latter case, the Football Association Premier League Ltd. Our proposed mechanism would scale up an idea that was once suggested for media firms [52] and applies it in areas where its benefits for biodiversity promise to be vastly superior. Assuming that biodiversity is a public good and that the above payment scheme should not be captured by private or sectoral interests, we propose that a scheme is established whereby companies would pay a fee to an existing or ad hoc institution representing the global public interest in preserving biodiversity, for using threatened animal representations in their branding. A voluntary program called “Save your logos” (http://www.saveyourlogo.org/) has been attempted, but we believe scaling up the idea requires grounding it in a formal compulsory mechanism. Global agreements such as the Convention on Biological Diversity (CBD) are often criticized for being ineffective due to their being non-compulsory and sometimes inexplicit [53]. The implementation of such payment mechanisms would be a major step towards improving their effectiveness to protect biodiversity.

The limits of so-called “standard economic” approaches to solve biodiversity conservation problems have been largely discussed in the literature [54,55]. The proposed mechanism should therefore not be seen as a panacea but rather as practical means to secure funding for conservation biology. The above critical analysis should also be used to identify and implement the important safeguards required to ensure proper implementation. These would include avoiding lobbying attempts to influence endangerment classification either way (from conservationists as well as from companies) and would address fairness as well as equity issues from the companies’ standpoints. Other perverse effects, such as attempts by companies to marginally modify representations to argue that they refer to fictitious rather than to real species, should also be listed and addressed. It would also probably be beneficial if the compensatory mechanism could be coupled with information campaigns from the companies about the reason for such funding, i.e., about the conservation status of their icons. This option would further benefit their conservation while possibly being better perceived by the company and their customers. Being perceived as acting at the forefront of the conservation of the imperiled charismatic animal that represents them could even create a very positive response from previous and new customers of such companies. Indeed, these firms may improve their corporate social responsibility by helping to save their icons, providing them with additional incentives to adhere to this scheme. Another critical element will be the choice of the institution(s) entrusted to receive the money and allot it to conservation initiatives. Global institutions devoted to biodiversity are prominent candidates, but other smaller-scale nonprofit local nongovernmental organizations (NGOs) might prove more resilient to interest groups and more knowledgeable about relevant local conservation issues in some situations. Elaborating an adequate institutional design and quantitatively calibrating the fees falls beyond the scope of the present paper and should be covered by interdisciplinary collaborations between conservation scientists and experts in the economic theory of incentives, institutional economics, and property right laws [55].

## Conclusion

Our study highlights that the 10 most charismatic animals for the public are in a dire conservation state but that the public is generally ignorant of this. Unless a radical change is operated, it is highly likely that most of these most-cherished species will go extinct in the wild within the next few decades. This situation is hidden by the large cultural abundance of these animals, which hinders conservation communication efforts and therefore acts as an additional, pernicious threat.

Beyond being a conservation tragedy in its own right, the likely extinction of these species can also turn into a double penalty for conservation biology. Indeed, charismatic species remain one of the most efficient vehicles to motivate the general public to support conservation action [56–58]. If these species go extinct in the wild, the whole conservation movement might suffer by losing its point in the eye of a large part of the general public.

We therefore claim that conservation studies, actions, and policies should stop seeing charismatic species as overprivileged conservation targets and face the fact that they are badly threatened species that urgently need an intensification of conservation effort. Such an intensification would not amount to demeaning the importance of conserving other elements of biodiversity, poorly known species, and whole ecosystems. For one thing, increasing the protection on the charismatic species does not mean zeroing on other conservation targets, especially if involved funding mechanisms are additive to already existing resources [53], as we propose here. Besides, because most charismatic species are keystone species with large habitat requirements, preserving them can have cascading co-benefits on the conservation status of numerous other species and ecosystem properties [59]. Similarly, communicating more about the endangerment of the most beloved species could raise public awareness of wider conservation issues.

Despite the fact that the increase needed in conservation revenue has been estimated to be more modest than other domains of public expenditure by at least one order of magnitude [60], in a world in which budget constraints are everywhere, our call for conservation intensification can look like wishful thinking. That is why we suggest, as a concrete mechanism to ensure its feasibility, a support from companies that use charismatic, endangered species for their branding. Setting up such a fund-raising scheme will require innovative interdisciplinary works involving conservation scientists, environmental economists, and legal scholars, but the relevant expertise is available, and action is required urgently.

## Supporting information

S1 Text (DOCX)

S2 Text (DOCX)

S1 Table Range size, proportion that is both suitable and protected (see S1 Text), and fragmentation (ratio of range size over perimeter of the range size) for the 10 most charismatic animals. (DOCX)

S1 Fig Ranking of the 10 most charismatic animals, according to the general public. These correspond to 13 species, as elephants and gorillas are represented by three and two species, respectively.
(TIF)

S2 Fig Number of sightings of each of the 10 animals in “virtual” populations (commercial, artistic, cultural, in zoos, books, magazines, on objects, on television, etc.) during 7 consecutive days by 42 volunteers living in France. Dark blue is the number of sightings and light blue is the total cumulative number of individuals seen (e.g., a chocolate bar with 1 elephant counts as 1 in dark blue and 1 in light blue, while a bar with 2 elephants counts as 1 in dark and 2 in light blue). Volunteers all lived in France but in various settings (from staying always indoors with no television in a rural house, to regular use of the internet and television and going out every day to work and shop in a large city). Volunteers were asked to pay attention to representation of those 10 animals in order to record them but not to seek them. After an information meeting, a one-day trial was used to homogenize observation behaviors and information recording.
(TIF)

S3 Fig Distribution of different drivers of threat for each of the 13 species, according to whether they are directly or indirectly human caused. Colors indicate whether a threat is primary (red) or secondary (yellow).
(TIF)

## Abbreviations

#patch: number of patches
CBD: Convention on Biological Diversity
Dyn: dynamical
IUCN: International Union for the Conservation of Nature
LC: Least Concern
MVP: minimum viable population
NGO: nongovernmental organization
