# Supplementary material for: The paradoxical extinction of the most charismatic animals
Source: PLoS Biol. 2018 Apr 12;16(4):e2003997. doi: 10.1371/journal.pbio.2003997 (PMC5896884; doi:10.1371/journal.pbio.2003997)
Supplement: S1 Text — (DOCX) [file pbio.2003997.s001.docx]

Materials and Methods

Charismatic species ranking

We used four different sources to assess which species the public found the most charismatic. First, an Internet survey was conducted through social networks using a specifically designed website, asking respondents about their choice of the 10 wild species they considered the most charismatic. The survey was available in English, French, Spanish and Italian and we collected answers from a total of 4,522 respondents from 69 countries. Second, the same survey was conducted with children ~10 years old in three primary schools in England, Spain and France. The schoolteachers first explained the term “charismatic” as attracting or appealing or preferred, and pupils were given 10-15 minutes to provide the 10 wild species. We collected 224 useable complete questionnaires. Third, we collected the names of all identifiable wild animal species displayed on the home page of the official web sites of major zoos from the 100 largest cities in the world (https://en.wikipedia.org/wiki/World's_largest_cities accessed on 16-04-2012). Fourth, we recorded the names of all identifiable animal species (excluding imaginary, extinct or domestic ones) featured on the cover of the English versions of all the animated movies ever produced by Disney and Pixar. Full details of the methods are given here [1]. We then combined the four lists into one, giving them equal weight, which provided our ranking of the most charismatic species.

Estimation of the values in Tables 1 and S1

The range size of each species has been calculated with the geospatial data provided by the IUCN [2]. In order to estimate the percentage of range coverage by protected areas, we combined species range size with data provided by Protected Planet that lists worldwide information on protected areas [3]. The index of fragmentation we proposed is simply the ratio of perimeter to range size of each species. In addition, we assessed suitable habitat in the global range size using LandCover data [4]. To do so, we separated natural from non-natural (anthropogenized) habitat and determined the corresponding surface. For these species, classes corresponding to anthropogenized or unavailable lands were 11, 14, 20, 30, 170, 190, 200, 210 and 220 (see [4] for details). All analyses have been conducted with QGis 2.18.1 and RStudio 0.99.853 software. For the latter, packages “geosphere”, “maptools”, “raster”, “rgdal”, “rgeos”, “proj4” and “shapefiles” have been used.

References:

1. Albert C, Luque GM, Courchamp F. The twenty most charismatic species. PLoS One. 2017;Submitted.

2. IUCN. The IUCN Red List of Threatened Species. In: 2017 [Internet]. [cited 4 Feb 2017]. Available: http://www.iucnredlist.org

3. IUCN, UNEP-WCMC. The World Database on Protected Areas (WDPA) [Internet]. 2016 [cited 4 Nov 2016]. Available: www.protectedplanet.net

4. Arino O, Perez R, Julio; J, Kalogirou V, Bontemps S, Defourny P, et al. Global Land Cover Map for 2009 (GlobCover 2009). 2012. doi:10.1594/PANGAEA.787668
