# Supplementary material for: The paradoxical extinction of the most charismatic animals
Source: PLoS Biol. 2018 Apr 12;16(4):e2003997. doi: 10.1371/journal.pbio.2003997 (PMC5896884; doi:10.1371/journal.pbio.2003997)
Supplement: S2 Text — (DOCX) [file pbio.2003997.s002.docx]

Supplementary Text

Conservation status

Our study shows that the ten most charismatic animals for the public are globally in a dire conservation status. Not only are their conservation statuses globally disastrous (Table 1), but they have dramatically worsened recently. Despite representing a blink in the time scale of species evolution, and notwithstanding being the most active and successful period of conservation biology, the last few decades have been catastrophic for almost all these species (see Fig. 1).

In addition, it is noteworthy that these low numbers of remaining animals tend to understate the actual threats on the species. These aggregated population sizes overlook the fact that most species are scattered among many isolated populations, and that a significant proportion of animals are not breeding and cannot contribute to the future of the species.

The proportion of breeding individuals in a population is highly variable among species. Among the 3,159 remaining tigers, it is estimated that there are fewer than 1,000 breeding females [1]. In addition, most tigers currently live in isolated patches that are too small to shelter demographically viable populations: all extant populations hold fewer than 100 breeding individuals [1,2]. Similarly, African lions live in 67 fragments of habitat, most of which is unprotected and heavily populated, and the projections for the next few decades show further decline and local extinctions [3]. In Africa, there were at least 219 isolated populations of elephants in the last assessment in 2008 [4]. Asian elephants are in fact three different subspecies dispersed into at least 138 patches over 13 countries [5]. For the giraffe, only about 70% of the individuals can be considered ‘mature’ for status assessment purposes [6]. Leopards survive on less than 25% of their historical range (less than 5% for four subspecies) and the remaining populations are scattered across 273 separate patches [7]. Mature adults are estimated to account for only 50.5% of the total population of pandas, so the actual reproductive population would be closer to 1,000 individuals in total [8]. They are in addition divided into 33 small, fragmented areas, corresponding to several disconnected populations of much fewer individuals, each with increased probability of extinction [9]. The estimated 7,000 remaining cheetahs are distributed across 30 subpopulations, 28 of which are estimated to hold fewer than 250 individuals [10,11], which indicates low survival probability in the future.

Another surprising result of this study is the finding that although the focus species are the most charismatic, a major threat faced by almost all of them is direct killing by humans (Fig. S3). IUCN threat categories show that poaching, conflict with humans, civil unrest, trophy hunting, diseases and car accidents account for over half of all the threats on the 12 species. This is certainly counterintuitive for species supposed to be among the most beloved ones.

Studies about the future of these species are surprisingly few and restricted, given their cultural importance. Most studies focus on one single threat and/or on one single population, making it difficult to get a global picture of the extinction risks of any of these species. However, even extinction projections based on a subset of threats show very low survival probabilities in the wild in the coming decades. Bengal tiger (*P. t. tigris*) population models indicate a 63-99% extinction likelihood within two to four decades due to poaching [12] while extinction probability due to inbreeding is as high as 90% within the next 30 years [13]. These two causes are also likely to result in the extinction of nearly all subpopulations of the Sumatran tiger (*P. t. sumatrae*) within the next 40 years [14]. Unsustainable bushmeat hunting, trophy hunting, habitat loss and human conflict all combine to make most of African lion populations surviving the next few decades unlikely [15–17]. Because of their long generation time, models indicate that without stringent conservation measures, elephants will become extinct within 20-100 years, both in Africa [18] and in Asia [19]. The Great Elephant Census (GEC) also predicts a likely extinction of savannah elephants in the wild before the end of the century [20]. Climate change is predicted to eliminate most of the panda's bamboo habitat in the next decades, leading to predictions of dramatic decline of the remaining panda populations [21,22]. Modelling different scenarios of global cheetah populations showed further population declines by 53% to 70% over the next 15 years and likely global extinction in the short term [11]. A forecast for polar bears worldwide concluded that they will further decline by two thirds by 2050 and that extinction is the most probable overall outcome for all but one population within 50 years [23]. This is mostly because of declines in sea ice extent following climate change, with a loss of optimal polar bear habitat that will reach 68% by the end of the century [24]. With the current annual rate of decline of 5%, Grauer’s gorillas will probably become extirpated from many parts of their range within 2-5 years [25], with about 93% of the total population gone in just three generations [26].

The average minimum viable population sizes for 8 out of the 10 animals (no estimate was available for either giraffes or leopards) were all below 5,800 individuals [27,28].

Despite these dire figures, these species are not exceptions: it is now common knowledge that biodiversity as a whole is impacted by human populations in a number of ways. For example, the 2016 Living Planet assessment report [29] revealed that 58% of wild animals have disappeared in the last four decades and that this loss would reach two thirds by 2020. According to the IUCN Red List, over 39% of all assessed species are threatened with extinction, and many taxonomic groups that have not been adequately assessed could be even more heavily threatened. A recent study on vertebrates showed that biodiversity loss is also acute at the population level [30]. Overall, nearly two thirds of the 27 world’s largest carnivores (≥15 kg) and of the 74 world’s largest herbivores (≥100 kg) are threatened with extinction [31,32]. Not all threatened species benefit from the conservation effort of the ten most charismatic animals, and many are at least as likely to be lost in a geologically extremely short time.

Lack of scientific knowledge

The above results should not hide another dreadful point: science knows incredibly little about these large, familiar and culturally ubiquitous species. In most cases, even basic information such as global population size is not known with certainty. Population sizes of lions and tigers have been the topic of heated debates among experts for years. The last estimates of the largest land mammal, elephant, are based on only 62% of its possible range: no population estimates are available for the remaining 38% [33]. Asian elephant population estimate (of 40-50,000) is qualified by experts as “no more than a crude guess”, dating from 2003, and yet is still the only available [5]. There are no reliable continent-wide estimates of leopard population size in Africa, and the most commonly cited estimate of over 700,000 leopards in Africa is known to be flawed [34]. In India, current estimates are based on pugmark censuses, a methodology which has been criticized as inaccurate [34]. For polar bears, nine out of 19 populations have insufficient data to provide an assessment of current trends and no information at all is available on abundance for the Arctic Basin population [35].

**References**

1. Walston J, Robinson JG, Bennett EL, Breitenmoser U, da Fonseca G a B, Goodrich J, et al. Bringing the tiger back from the brink-the six percent solution. PLoS Biol. 2010;8: 6–9. doi:10.1371/journal.pbio.1000485

2. Ranganathan J, Chan KMA, Karanth KU, Smith JLD. Where can tigers persist in the future? A landscape-scale, density-based population model for the Indian subcontinent. Biol Conserv. 2008;141: 67–77. doi:10.1016/j.biocon.2007.09.003

3. Riggio J, Jacobson A, Dollar L, Bauer H, Becker M, Dickman A, et al. The size of savannah Africa: A lion’s (Panthera leo) view. Biodivers Conserv. 2013;22: 17–35. doi:10.1007/s10531-012-0381-4

4. Blanc J. Loxodonta africana. IUCN Red List Threat Species 2008. 2008;8235. doi:http://dx.doi.org/10.2305/IUCN.UK.2008.RLTS.T12392A3339343.en

5. Choudhury A, Lahiri Choudhury DK, Desai A, Duckworth JW, Easa PS, Johnsingh AJT, et al. Elephas maximus. IUCN Red List Threat Species 2010. 2008; doi:e.T7140A12828813

6. Muller Z, Bercovitch F, Brand R, Brown D, Brown M, Bolger D, et al. Giraffa camelopardalis. IUCN Red List Threat Species 2016. 2016; 1–8. doi:10.1126/science.194.4268.933

7. Jacobson AP, Gerngross P, Lemeris Jr. JR, Schoonover RF, Anco C, Breitenmoser-Würsten C, et al. Leopard (Panthera pardus) status, distribution, and the research efforts across its range. PeerJ. 2016;4: e1974. doi:10.7717/peerj.1974

8. Swaisgood R, Wang D, Wei F. Ailuropoda melanoleuca. IUCN Red List Threat Species 2016. 2016; doi:10.2307/3503982

9. Kang D, Li J. Premature downgrade of panda’s status. Science (80- ). 2016;354: 295–295. doi:10.1126/science.aaj1963

10. Durant S, Mitchell N, Ipavec A, Groom R. Acinonyx jubatus, cheetah. IUCN Red List Threat Species 2015. 2015; doi:10.1644/1545-1410(2005)771[0001:AJ]2.0.CO;2

11. Durant SM, Mitchell N, Groom R, Pettorelli N, Ipavec A, Jacobson AP, et al. The global decline of cheetah Acinonyx jubatus and what it means for conservation. Proc Natl Acad Sci. 2016;114: 201611122. doi:10.1073/pnas.1611122114

12. Horev A, Yosef R, Tryjanowski P, Ovadia O. Consequences of variation in male harem size to population persistence: Modeling poaching and extinction risk of Bengal tigers (Panthera tigris). Biol Conserv. 2012;147: 22–31. doi:10.1016/j.biocon.2012.01.012

13. Kenney J, Allendorf FW, Mcdougal C, Smith JLD. How much gene flow is needed to avoid inbreeding depression in wild tiger populations ? Proc R Soc. 2014;281: 20133337.

14. Linkie M, Chapron G, Martyr DJ, Holden J, Leader-Williams N. Assessing the viability of tiger subpopulations in a fragmented landscape. J Appl Ecol. 2006;43: 576–586. doi:10.1111/j.1365-2664.2006.01153.x

15. Creel S, M’soka J, Dröge E, Rosenblatt E, Becker M, Matandiko W, et al. Assessing the sustainability of African lion trophy hunting, with recommendations for policy. Ecol Appl. 2016; doi:10.1002/eap.1377

16. Bauer H, Chapron G, Nowell K, Henschel P, Funston P, Hunter LTB, et al. Lion (Panthera leo) populations are declining rapidly across Africa, except in intensively managed areas. Proc Natl Acad Sci U S A. 2015;112: 14894–9. doi:10.1073/pnas.1500664112

17. Riggio J, Caro T, Dollar L, Durant SM, Jacobson AP, Kiffner C, et al. Lion populations may be declining in Africa but not as Bauer et al. suggest. Proc Natl Acad Sci. 2015;113: 201521506. doi:10.1073/pnas.1521506113

18. Lopes AA. Organized crimes against nature: Elephants in southern africa. Nat Resour Model. 2015; 86–107. doi:10.1111/nrm.12058

19. Goswami VR, Vasudev D, Oli MK. The importance of conflict-induced mortality for conservation planning in areas of human-elephant co-occurrence. Biol Conserv. Elsevier Ltd; 2014;176: 191–198. doi:10.1016/j.biocon.2014.05.026

20. Chase MJ, Schlossberg S, Griffin CR, Bouché PJC, Djene SW, Elkan PW, et al. Continent-wide survey reveals massive decline in African savannah elephants. PeerJ. 2016;4: e2354. doi:10.7717/peerj.2354

21. Li R, Xu M, Wong MHG, Qiu S, Li X, Ehrenfeld D, et al. Climate change threatens giant panda protection in the 21st century. Biol Conserv. Elsevier Ltd; 2015;182: 93–101. doi:10.1016/j.biocon.2014.11.037

22. Tuanmu M-N, Viña A, Winkler J a., Li Y, Xu W, Ouyang Z, et al. Climate-change impacts on understorey bamboo species and giant pandas in China’s Qinling Mountains. Nat Clim Chang. Nature Publishing Group; 2013;3: 249–253. doi:10.1038/nclimate1727

23. Amstrup SC, Marcot BG, Douglas DC. A Bayesian Network Modeling Approach to Forecasting the 21st Century Worldwide Status of Polar Bears. Geophys Monogr. 2008;180: 213–268.

24. Durner G, Douglas D, Nielson R, Amstrup S, McDonald T, Stirling I, et al. Predicting 21st-century polar bear habitat distribution from global climate models. Ecol Monogr. 2009;79: 25–58. doi:10.1890/07-2089.1

25. Plumptre AJ, Nixon S, Vieilledent G, Nishuli R, Kirkby A, Williamson EA, et al. Status of Grauer’s Gorilla and Chimpanzees in Eastern Democratic Republic of Congo: Historical and Current Distribution and Abundance. New York; 2015.

26. Plumptre A, Robbins M, Williamson EA. Gorilla beringei. IUCN Red List Threat Species 2016. 2016;

27. Reed DH, O’Grady JJ, Brook BW, Ballou JD, Frankham R. Estimates of minimum viable population sizes for vertebrates and factors influencing those estimates. Biol Conserv. 2003;113: 23–34. doi:10.1016/S0006-3207(02)00346-4

28. Traill LW, Bradshaw CJA, Brook BW. Minimum viable population size: A meta-analysis of 30 years of published estimates. Biol Conserv. 2007;139: 159–166. doi:10.1016/j.biocon.2007.06.011

29. World Wildlife Fund. Living Planet Report 2016 : Risk and resilience in a new ear. 2016.

30. Ceballos G, Ehrlich PR, Dirzo R. Biological annihilation via the ongoing sixth mass extinction signaled by vertebrate population losses and declines. Proc Natl Acad Sci. 2017; 1–8. doi:10.1073/pnas.1704949114

31. Ripple WJ, Newsome TM, Wolf C, Dirzo R, Everatt KT, Galetti M, et al. Collapse of the world’s largest herbivores. Sci Adv. 2015;1: e1400103–e1400103. doi:10.1126/sciadv.1400103

32. Ripple WJ, Estes J a, Beschta RL, Wilmers CC, Ritchie EG, Hebblewhite M, et al. Status and ecological effects of the world’s largest carnivores. Science (80- ). 2014;343: 1241484. doi:10.1126/science.1241484

33. Thouless CR, Dublin HT, Blanc JJ, Skinner DP, Daniel TE, Taylor RD, et al. African Elephant Status Report 2016. An update from the African Elephant Database. 2016.

34. Henschel P, Hunter L, Breitenmoser U, Purchase N, Packer C, Khorozyan I, et al. Panthera pardus. IUCN Red List Threat Species 2008. 2008;

35. Wiig O, Atwood T, Laidre K, Lunn N, Obbard M, Regehr E, et al. Ursus maritimus. IUCN Red List Threat Species 2015. 2015; doi:e.T22823A14871490.
