# Supplementary material for: The paradoxical extinction of the most charismatic animals
Source: PLoS Biol. 2018 Apr 12;16(4):e2003997. doi: 10.1371/journal.pbio.2003997 (PMC5896884; doi:10.1371/journal.pbio.2003997)
Supplement: S1 Table — (DOCX) [file pbio.2003997.s003.docx]

Table S1.

Range size, proportion that is both suitable and protected (see Material and Methods), and fragmentation (ratio of range size over perimeter of the range size), for the 10 most charismatic animals.
