# Supplementary figures and images for: The paradoxical extinction of the most charismatic animals
Source: PLoS Biol. 2018 Apr 12;16(4):e2003997. doi: 10.1371/journal.pbio.2003997 (PMC5896884; doi:10.1371/journal.pbio.2003997)

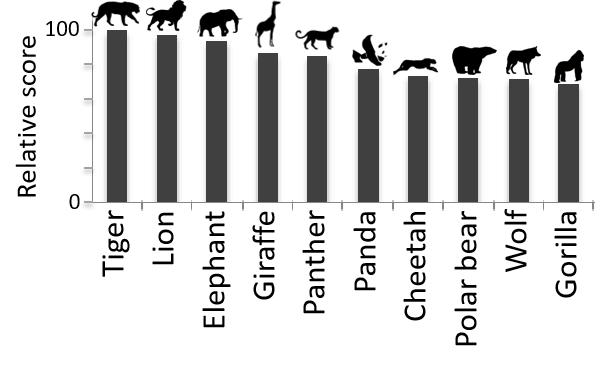

Supplement: S1 Fig — These correspond to 13 species, as elephants and gorillas are represented by three and two species, respectively. (TIF) [file pbio.2003997.s004.tif]

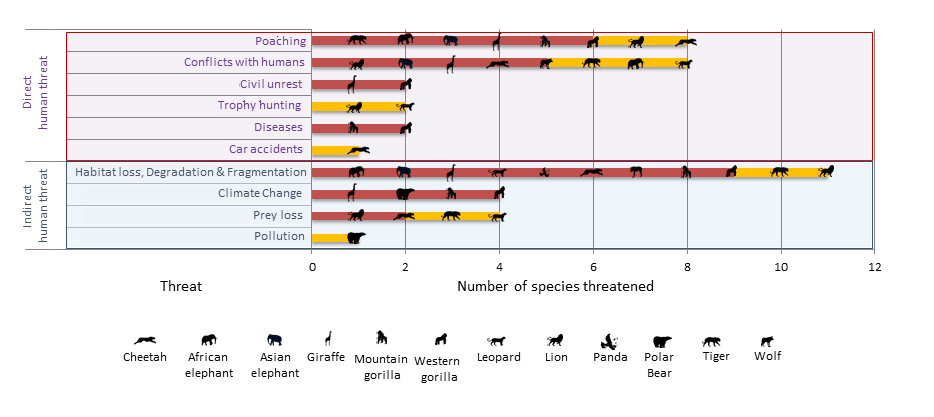

Supplement: S2 Fig — Dark blue is the number of sightings and light blue is the total cumulative number of individuals seen (e.g., a chocolate bar with 1 elephant counts as 1 in dark blue and 1 in light blue, while a bar with 2 elephants counts as 1 in dark and 2 in light blue). Volunteers all lived in France but in various settings (from staying always indoors with no television in a rural house, to regular use of the internet and television and going out every day to work and shop in a large city). Volunteers were asked to pay attention to representation of those 10 animals in order to record them but not to seek them. After an information meeting, a one-day trial was used to homogenize observation behaviors and information recording. (TIF) [file pbio.2003997.s005.tif]

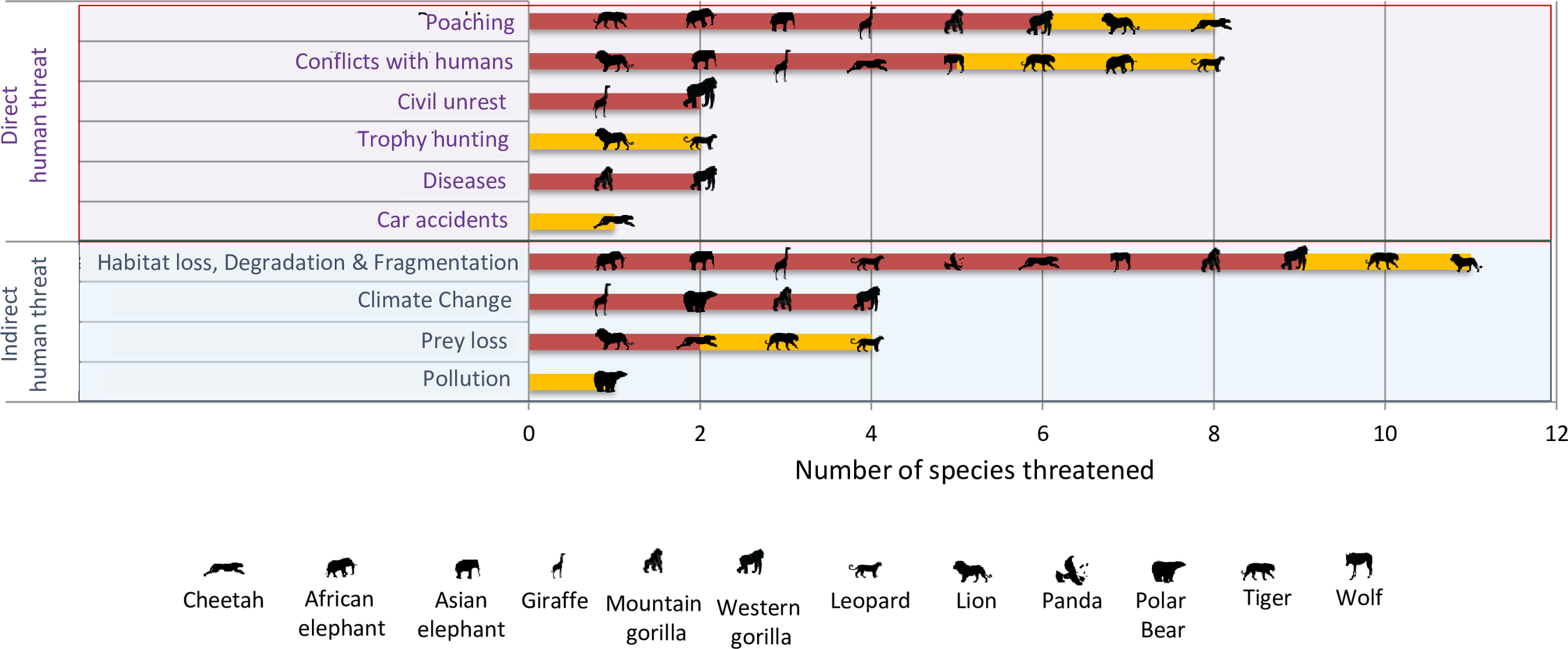

Supplement: S3 Fig — Colors indicate whether a threat is primary (red) or secondary (yellow). (TIF) [file pbio.2003997.s006.tif]
